# EBV-regulated global changes in mRNA isoform usage

**DOI:** 10.1186/1750-9378-7-S1-O3

**Published:** 2012-04-19

**Authors:** Nicholas Homa, Raul Salinas, Eleonora Forte, Timothy Robinson, Mariano Garcia-Blanco, Micah Luftig

**Affiliations:** 1 Department of Molecular Genetics and Microbiology, Duke University, Durham, NC, USA

## 

Approximately 90% of the global adult population is latently infected with Epstein-Barr Virus (EBV). Latent EBV infections are normally asymptomatic due to a robust cytotoxic T cell response. However, in the event of immunosuppression, as observed in HIV/AIDS patients, these latent infections can lead to B-cell lymphomas. *In vitro* EBV has the capacity to transform primary B-cells into immortalized Lymphoblastoid Cell Lines (LCLs). In order to assess changes in both overall mRNA abundance and mRNA isoform usage, we queried resting, primary human B cells and LCLs using Human Exon (HuEx) and conventional Affymetrix U133 arrays. Using a novel computational algorithm, SplicerEX, we identified 433 genes whose mRNAs undergo changes in alternative isoform usage during the transformation from primary B-cells to LCLs. Isoform changes were largely orthogonal from expression changes as only ~1/3 of mRNA isoform changes were also changed at the level of overall abundance. Isoform changes were classified into alternative 5’ initiation, internal exclusion/inclusion of exons, 3’ terminal exon choice, and 3’UTR alterations. The most striking mRNA isoform change was 3’UTR shortening, accounting for ~25% of all changes. Gene ontology analysis of mRNA isoform changes revealed a strong enrichment for nucleic acid binding proteins, including splicing and transcription factors. We have confirmed a subset of the predictions made by SplicerEX using isoform-specific RT-PCR. Importantly, many mRNA isoform changes observed were in fact regulated by EBV latent infection, not just proliferation per se, as they were also observed in the conversion of EBV-negative Burkitt’s lymphoma cells (BL41) to latency III expressing BL41/B95-8 cells. Our preliminary results further indicate that two transcription factors, the E2 family member TCF4 and the plasma cell differentiation factor XBP1, are both regulated by EBV at the level of alternative isoform usage. These proteins both impact activation of the BZLF1 lytic promoter and our data thus suggest a novel mechanism by which EBV maintains latent infection in immortalized B cells. These data may point to new approaches in regulating the latent/lytic switch crucial to the pathogenesis of EBV-associated AIDS lymphomas.

